# Percutaneous coronary artery intervention in unprotected left main coronary artery disease: one-year outcome Egyptian registry

**DOI:** 10.1186/s43044-022-00302-9

**Published:** 2022-09-06

**Authors:** Rana Ayman, Sameh Mohamed Shaheen, Sameh Saleh Sabet, Yasser A. Abdellatif

**Affiliations:** Cardiology Department, Ain Shams University Hospital, Nargess 3 – Fifth Settlement, Abbassya, PO 11835, Cairo, Egypt

**Keywords:** Left main artery, Bifurcation stenting, Provisional stenting, Two-stent technique

## Abstract

**Background:**

Left main coronary artery lesions are associated with jeopardy of an outsized area of the myocardium, causing a high incidence of morbidity and mortality. Optimal treatment of coronary bifurcation anatomy remains highly debatable, whether by provisional or two-stent technique. This prospective observational study was designed to investigate the one-year clinical outcomes of unprotected left main coronary artery disease revascularization by percutaneous coronary intervention in a “real-world” setting among Egyptian patients in a prospective single-center registry (at Ain Shams University Hospitals).

**Results:**

This study included 163 patients who underwent PCI to LM lesions between May 1, 2020, and the end of April in Ain Shams University hospitals. Patients were dichotomized into two groups according to their intended stenting technique, whether provisional or two-stent technique. A total of 142 underwent provisional stenting while 21 were designated for the two-stent technique, mainly DK crush (double kissing). Among the patients with intended provisional stenting, 34 patients underwent the TAP technique. Patients were followed up for the primary endpoints, at the in-hospital setting, at 30 days, and after 1 year. In-hospital death was encountered in 6.34% of cases undergoing provisional stenting, among which 5.36% were due to a cardiovascular cause. Total MACCE was found to be 2.96% in the provisional stenting group versus 4.76% in the two-stent group. Overall, MACCE at 1 year was found to be 22.31% in the provisional group and 30% in the two-stent group (*p*-value0.57). TVF was recognized in 10% of cases treated by provisional stenting and 30% of cases treated by the two-stent technique (*p*-value 0.023).

**Conclusions:**

LM coronary artery lesions treatment by PCI is considered a safe and beneficial solution. Provisional stenting is the preferred approach bearing in mind that bail-out procedures may be sought in case the SB needs further treatment. Adjunctive assessment by IVUS or FFR may help achieve better outcomes, and efforts should be performed to facilitate their feasibility.

## Background

Left main coronary artery (LMCA) lesions are associated with jeopardy of an outsized area of the myocardium, causing a high incidence of morbidity and mortality [1].

Significant LM disease is found in around 5% of patients who underwent invasive coronary angiography. The mortality of patients with LM is 2–3 times more than patients with one- or two-vessel disease. A higher degree of stenosis increases the risk [2].

Coronary artery bypass graft (CABG) has been the mainline revascularization strategy for LMCA disease [3]. However, with the improvement in PCI techniques and equipment, PCI certainty has expanded its role in left main revascularization over the past decade [4]. Percutaneous treatment of unprotected LM coronary artery disease evolved over time and currently is accepted as an alternate treatment to coronary artery bypass grafting (CABG) in certain patients where percutaneous coronary intervention (PCI) with drug-eluting stents (DES) has been demonstrated to be feasible and safe at midterm clinical follow-up [5–11].

Optimal treatment of coronary bifurcation anatomy remains highly debatable, whether by provisional stenting or two-stent technique [12]. Trials of bifurcation lesions have demonstrated that there's no superiority to systematic two-stent strategies [12–14] and that long-term mortality would be worse with a more complex approach [15]. For the LM stem, it might be expected that these differences would be more encountered due to the wide angle of separation between the 2 vessels, the heavy calcification often present, and the undeniable fact that neither vessel is a side branch, but both are rather important in most cases. Non-randomized data usually suggest that outcomes are worse with a two-stent technique [16–18], but randomized data support the double kissing (DK)-crush technique for true bifurcation left main stem disease [19, 20].

## Methods

This prospective observational study was designed to investigate the one-year clinical outcomes of unprotected left main coronary artery disease revascularization by percutaneous coronary intervention in a “real-world” setting among Egyptian patients in a prospective single-center registry (at Ain Shams University Hospitals). It aims at exploring the trend of LM PCI at our cardiac cath laboratory. The aim is to express our outcomes and the extent of correlation with international centers.

### Study population

The study was conducted within the period from May 1, 2020, to the end of April 2021, including patients diagnosed with LM disease who underwent LM percutaneous intervention. All of them were recruited from the department of cardiovascular medicine at Ain Shams University Hospitals. Patients' participation was voluntary, and the subject had the right to withdraw from the study at any time without affecting his/her further medical care. Patients were considered eligible for the study if they were symptomatic, whether as a chronic coronary syndrome or acute coronary syndrome (including acute STEMI) and underwent PCI to a significant unprotected LM disease (whether located in the ostium, shaft, or distal). Significant LM disease was defined as visually assessed stenosis diameter ≥ 50%, fractional flow reserve ≤ 0.80, or documented stress-induced myocardial ischemia on non-invasive imaging. The patient was assigned to the percutaneous revascularization modality at the operator's discretion considering each patient's SYNTAX and STS scores in agreement with the 2018 ESC guidelines for myocardial revascularization [1]. Patients with high SYNTAX scores were discussed by the heart team, and the decision to resort to PCI was due to high surgical risk, surgical ineligibility, or the patient's refusal to undergo CABG.

The main exclusion criteria in the study were protected left main, acute ST-elevation myocardial infarction with cardiogenic shock, chronic total occlusion of either LAD and/or LCX, PCI done using bare-metal stents, severely impaired left ventricular ejection fraction (LVEF ≤ 20%), patients indicated for concomitant valvular or aortic surgery or patient life expectancy below 1 year. Approval was obtained from the ethical committee at Ain Shams University before starting the research. (FWA 000017585).

Preprocedural data were collected from the patients regarding their presentation (chronic coronary syndrome or acute coronary syndrome), comorbidities (peripheral arterial disease, chronic lung disease, chronic kidney disease), and risk factors (smoking, diabetes mellitus, hypertension, dyslipidemia, positive family history, obesity assessed by body mass index). Laboratory data were collected, including cardiac enzymes, glycated hemoglobin and serum LDL. ECG and echocardiography to estimate left ventricular ejection fraction (using the Modified Simpson method) were collected as well. During the procedure, intravenous unfractionated heparin 70 IU/kg was given at the beginning of the procedure to keep an activated clotting time of > 250 s. Access site, use of glycoprotein inhibitors, and use of IVUS or FFR were at the discretion of the operator. All patients were on acetyl salicylic acid (aspirin) and P2Y12 inhibitor (either clopidogrel or ticagrelor).

The choice of stenting technique was at the operator's discretion considering the European Bifurcation Club (EBC) recommendations [2].

### Stepwise provisional single-stent group

Coronary guidewires were passed to the left anterior descending (LAD) and circumflex (LCx) arteries, respectively. Either the LAD or LCx will be designated as the main branch (MB), and the other will be the side branch (SB). Lesion preparation was undertaken as needed to be followed by stenting of the LAD, a wire jailed in the LCx to preserve side vessel flow and access. Following stenting of the left main into the LAD, proximal optimization (POT) of the left main artery part of the stent was encouraged to be done. This was occasionally followed by rewiring the LCX, opting for a distal cell crossing, and kissing balloon inflations that may eventually be followed by POT.

Angiographic success was considered if the following were absent: < TIMI 3 flow in the side vessel, severe (> 90%) ostial pinching of the SB or SB dissection. Under these circumstances, the operator resorted to implanting an SB stent in a manner of their choosing (e.g., TAP).

The TAP stenting technique is a modification of the T-stenting technique designed to optimize "bail-out" SB stent implantation in bifurcation lesions treated by the "provisional" approach. It is applied after the MB stent has been implanted and kissing balloon inflation has been performed. In particular, TAP stenting was developed to ensure full ostium coverage by DES struts while requiring the performance of final kissing inflation. For this to succeed, the SB stent is delivered with minimal protrusion inside the MB with an uninflated balloon positioned in the MB across the SB take-off. After SB stent deployment, kissing balloon inflation is immediately performed with the stent's balloon and the balloon which was previously positioned in the MB [21].

### Systematic, planned two-stent group

Coronary guidewires were passed to the LAD and LCx/intermediate arteries. Pre-stenting balloon inflation was performed. The stent technique was at the discretion of the operator but could be one of culotte, DK crush, minicrush, or TAP. Steps varied according to the technique chosen [22]. However, no planned culotte technique was done in our study.

The DK-crush technique consists of stenting the SB, balloon crush, first kissing, stenting the MB, and final kissing balloon inflation. Careful rewiring from the proximal cell of the MB stent and keeping the wire in the true lumen of the SB stent are very important for optimal angiographic results. Balloon anchoring from the MB, alternative inflation, and each kissing inflation using large enough noncompliant balloons at high pressure and the POT technique are mandatory to improve both angiographic and clinical outcomes [23].

When describing the minicrush technique, pre-dilatation of all branches was performed with balloon kissing of both the MB and the SB at first, then between the MB and the proximal branch afterward. This was followed by the positioning of stents in both side branches (SB stent's proximal dot was situated in the MB at a length of 1–2 mm proximally to the carina of the bifurcation). SB stents were then deployed sequentially and crushed at the same time by high-pressure balloon inflation positioned in the MB [24].

#### Post-procedural

Aspirin 75–100 mg daily was continued long-term in addition to a P2Y12 inhibitor, whether clopidogrel or ticagrelor, according to the operator's choice. Statin therapy was continued for the duration of the study, whether rosuvastatin or atorvastatin.

#### Follow-up

The primary endpoint of the study was a composite of all-cause death and four-point major adverse cardiovascular and cerebrovascular events (MACCE) during the 12 months follow-up, including cardiovascular mortality, myocardial infarction, and cerebrovascular accidents (TIA, embolic or hemorrhagic stroke), and hospitalization for unstable angina or revascularization procedures.

The Universal Definition of Myocardial Infarction was used to define myocardial infarction in this study. Coronary intervention-related MI (MI type 4a) is defined by the rise of cTn values more than five times the 99th percentile upper reference limit (URL) in patients with normal baseline values. In patients with an increased pre-procedure cTn in whom the cTn levels are stable (≤ 20% change) or falling, the post-procedure cTn must rise by > 20%. However, the absolute post-procedural increase must still be at least five times the 99th percentile URL. In addition to that, one of the following elements is mandatory: new-onset ischemic ECG changes; development of new pathological Q waves; or angiographic findings indicating a procedural flow-limiting complication such as coronary dissection, occlusion of a major epicardial artery, or an SB occlusion/thrombus, disruption of collateral flow or distal embolization [25].

Technical success is defined as completion of stent placement, balloon dilatation, rewiring, and final kissing balloon therapy as required by the protocol. Procedure success is defined as the placement of stents as per randomization with TIMI 3 flow and < 30% stenosis in any stented vessel and TIMI 3 flow in any unstented vessel [22].

Target vessel failure (TVF) is defined as a combined endpoint by the presence of re-occlusion, restenosis, or target vessel revascularization (defined as a necessity for a repeated PCI within the formerly intervened vessel) [26].

#### Statistical analysis

The collected data were revised, coded, tabulated, and introduced to a PC using the Statistical Package for Social Science (SPSS 25). Data were presented, and suitable analysis was done according to the type of data obtained for each parameter. *Student t-test* was used to assess the statistical significance of the difference between the two study group parametric variables, and the Mann–Whitney test (*U*) test was used for nonparametric variables. For categorical variables, *the Chi-square test* was used, while *Fisher's exact test* was used when the expected count is less than 5 in more than 20% of cells.

## Results

This is a descriptive study that recruited 163 patients who underwent PCI for LM lesions. Patients were dichotomized into two groups according to their intended stenting technique, whether provisional or two-stent technique. A total of 142 underwent provisional stenting while 21 were designated for the two-stent technique, mainly DK crush. Among the patients with intended provisional stenting, 34 patients underwent the TAP technique.

Patients were followed up for the primary endpoints, at the in-hospital setting, at 30 days, and after 1 year.

### Patients' characteristics and clinical features

Both groups were not significantly different as regards age, smoking status, covid status, family history of ischemic heart disease, or body mass index (BMI); however, a significantly higher proportion of males were represented in the two-stent technique group (81%) compared to that in the provisional group (55%). There was no significant difference between both groups regarding risk factors such as DM, HTN, CKD, CLD, or PAD (Table 1).

**Table 1:** Patient’s characteristics and clinical features

	Intended stenting technique		Test of significance		
	Provisional	Two-stent technique			
	Mean ± SD *N* (%)	Mean ± SD *N* (%)	Value	*p*-Value	Sig
Age	58.61 ± 9.5	61.29 ± 8.5	***t* = − **1.219	0.225	NS
Sex					
Male	78 (54.93)	17 (80.95%)	***X***^***2***^** = **5.095	0.024	S
Female	64 (45.07)	4 (19.05%)			
Smoking	55 (38.73)	11 (52.38)	***X***^***2***^** = **1.414	0.234	NS
BMI					
Normal	30 (21.13)	7 (33.33%)	Fisher's exact test	0.602	NS
Overweight	50 (35.21)	7 (33.33%)			
Obese 1	48 (33.8)	5 (23.81%)			
Obese 2	14 (9.86)	2 (9.52%)			
FH					
Negative	108 (76.06)	12 (57.14%)	***X***^***2***^** = **3.37	0.066	NS
Positive	34 (23.94)	9 (42.86%)			
COVID status					
Negative	137 (96.48)	19 (90.48%)	Fisher's exact test	0.223	NS
Positive	5 (3.52)	2 (9.52%)			
DM	96 (67.61%)	15 (71.43)	***X***^***2***^** = **0.123	0.726	NS
HTN	106 (74.65%)	13 (61.9)	***X***^***2***^** = **1.507	0.22	NS
CKD	33 (23.24%)	2 (9.52)	Fisher's exact test	0.253	NS
Chronic liver disease	11 (7.75%)	1 (4.76)	Fisher's exact test	1.00	NS
(PAD)Lower extremity	21 (14.79%)	2 (9.52)	Fisher's exact Test	0.248	NS
(PAD)Carotid	1 (0.7%)	1 (4.76)			
(PAD)Mesenteric or renal	0 (0%)	0 (0)			

*Chi-square test of significance (*X*^2^)

Laboratory data such as hemoglobin level, creatinine clearance, pre- and post-procedural cardiac enzymes, and HbA1c and LDL did not show any difference of significant value between the two groups. Patients were mostly of EF < 40%, with an incidence of 42.25% and 61.9% in the provisional stenting and the two-stent technique groups, respectively. Most patients were in sinus rhythm as well. Both variables were of no statistical significance (Table 2).

**Table 2:** Laboratory data, ECG and cardiac functions

	Intended stenting technique		Test of significance		
	Provisional	Two-stent technique			
	*N* (%) Mean ± SD Median (IQR)	*N* (%) Mean ± SD Median (IQR)	Value	*p*-Value	Sig
Dyslipidaemia	97 (68.31)	16 (76.19)	***X***^***2***^** = **0.534	0.465	NS
Hemoglobin	12.75 ± 2.37	13.28 ± 1.28	***t* = − **1.554	0.127	NS
Creatinine clearance					
> 90	104 (73.24)	21 (100)	Fisher's exact test	0.088	NS
60–89	19 (13.38)	0 (0)			
30–59	17 (11.97)	0 (0)			
15–29	1 (0.7)	0 (0)			
< 15	1 (0.7)	0 (0)			
HbA1c					
Normal	40 (28.17)	4 (19.05)	***X***^***2***^** = **1.272	0.529	NS
Pre-diabetic	12 (8.45)	3 (14.29)			
DM	90 (63.38)	14 (66.67)			
LDL	130 (100–190)	160 (90–170)	***z* = − **0.117	0.907	NS
EF					
> 50%	49 (34.51)	3 (14.29)	***X***^***2***^** = **3.925	0.141	NS
> 40%	60 (42.25)	13 (61.9)			
40–49%	33 (23.24)	5 (23.81)			
ECG rhythm					
Sinus	139(97.89)	21 (100)	Fisher's exact test	0.537	NS
AF	2 (1.41)	0 (0)			
CHB	1 (0.7)	0 (0)			

*Chi-square test of significance (*X*^2^)

*Student *t*-test of significance (*t*)

*Mann–Whitney test of significance (*z*)

### Procedural and technical data (Table 3)

**Table 3:** Procedural and technical data

	Intended stenting technique		Test of significance		
	Provisional	Two-stent technique			
	*N* (%)	*N* (%)	Value	*p*-Value	Sig
*Presentation*					
CCS	43 (30.28)	6 (28.57)	Fisher's exact test	0.036	S
UA	22 (15.49)	4 (19.05)			
NSTEMI	33 (23.24)	9 (42.86)			
STEMI	42 (29.58)	1 (4.76)			
Post-arrest	2 (1.41)	1 (4.76)			
*No. of vessels affected*					
1(Isolated LM)	6 (4.23)	0 (0)	Fisher's exact test	0.02	S
2(LM + 1vessel)	79 (55.63)	7 (33.33)			
3(LM + 2vessel)	44 (30.99)	14 (66.67)			
4(LM + 3vessel)	13 (9.15)	0 (0)			
*Distribution of bifurcation*					
LM	6 (4.23)	0 (0)	Fisher's exact test	< 0.001	S
LM_LAD	109 (76.76)	2 (9.52)			
LM_LCX	2 (1.41)	0 (0)			
LM_LAD_LCX	25 (17.61)	19 (90.48)			
*Preparation of MB*					
yes	108 (76.06)	19 (90.48)	Fisher's exact test	0.168	NS
*Preparation of SB*					
yes	34 (23.94)	19 (90.48)	Fisher's exact test	< 0.001	S
*Medina classification*					
1,1,1	30 (21.13)	19 (90.48)	Fisher's exact test	< 0.001	S
1,1,0	75 (52.82)	2 (9.52)			
1,0,1	28 (19.72)	0 (0)			
0,1,1	5 (3.52)	0 (0)			
1,0,0	2 (1.41)	0 (0)			
0,1,0	2 (1.41)	0 (0)			
*Vessel stented first*					
MB	142 (100)	0 (0)	Fisher's exact test	< 0.001	S
SB	0 (0)	21 (100)			
*TIMI flow in SB after 1st stent*					
0-I	3 (2.11)	0 (0)	Fisher's exact test	0.833	NS
II	21 (14.79)	4 (19.05)			
III	118 (83.1)	17 (80.95)			
*Rewiring the SB*					
No	38 (26.76)	0 (0)	Fisher's exact test	0.004	S
Yes	104 (73.24)	21 (100)			
*Kissing balloons after 1st stent*					
Yes	99 (69.72)	20 (95.24)	*X*^***2***^** = **6.046	0.014	S
*Further improvement to SB needed*					
Yes	31 (21.83)	21 (100)	*X*^***2***^** = **51.46	< 0.001	S

	Intended stenting technique		Test of significance		
	Provisional	Two-stent technique			
	*N* (%) Median (IQR) Mean ± SD	*N* (%) Median (IQR) Mean ± SD	Value	*p*-Value	Sig
Reason for the second stent					
Residual lesion	11 (7.75)	21 (100)	Fisher's Exact test	< 0.001	S
Vessel compromise	19 (13.38)	0 (0)			
Dissection inside vessel	4 (2.82)	0 (0)			
Kissing balloons after 2nd stent					
Yes	28 (19.72)	20 (95.24)	***X***^***2***^** = **50.22	< 0.001	S
Final POT					
Yes	126 (88.73)	21 (100)	Fisher's Exact test	0.229	NS
Eventual technique					
Provisional	108 (76.06)	0 (0)	Fisher's Exact test	< 0.001	S
Provisional to double	34 (23.94)	0 (0)			
Two-stent	0 (0)	21 (100)			
Two-stent technique					
Provisional	108 (76.06)	0 (0)	Fisher's Exact test	< 0.001	S
Minicrush	0 (0)	3 (14.29)			
DK crush	0 (0)	18 (85.71)			
TAP	34 (23.94)	0 (0)			
GPIIb/IIIa inhibitors					
Yes	25 (17.61)	1 (4.76)	Fisher's Exact test	0.203	NS
MB stent diameter	3.5 ± 0.39	3.63 ± 0.3	***t* = − **1.488	0.139	NS
MB stent length	32.32 ± 7.74	29.52 ± 6.83	***t* = **1.566	0.119	NS
SB Stent					
Yes	34(23.94)	21 (100)	***X***^***2***^** = **48.66	< 0.001	S
SB stent diameter	3.33 ± 0.35	3.23 ± 0.31	***t* = **1.111	0.272	NS
SB stent length	23.15 ± 4.21	24.95 ± 8.58	***t* = − **0.896	0.378	NS
Syntax score	25.18 ± 6.12	29.81 ± 4.13	***t = − ***4.466	< 0.001	S
STS score	1.4 (0.8–2.1)	1.2 (0.9–2.2)	***z* = − **0.404	0.686	NS
Use of IVUS or FFR					
Yes	9 (6.34)	0 (0)	Fisher's exact test	0.606	NS

	Provisional		Two-stent technique		Fisher exact test	
	*N*	%	*N*	%	*p*-value	sig
Additional vessel stented	36	25.4	1	4.8	0.047	S
RCA	26	18.3	0	0.0	0.027	S
OM	5	3.5	0	0.0	1	NS
Diagonal	3	2.1	1	4.8	0.427	NS
LAD	2	1.4	0	0.0	1	NS
LCX	2	1.4	0	0.0	1	NS

*Chi-square test of significance (*X*^2^)

*Mann–Whitney test of significance (*z*)

*Student t-test of significance (*t*)

The most common presentation for patients undergoing provisional stenting was chronic coronary syndrome (30.28%), but it was NSTEMI (42.86%) for patients treated by the two-stent technique (*p*-value 0.036).

Syntax scores were 25.18 ± 6.12 in the provisional stenting group and 29.81 ± 4.13 in the two-stent technique group (*p*-value < 0.001). STS ranged from 0.8 to 2.1% in the provisional stenting group and from 0.9 to 2.2% in the two-stent technique group (*p*-value = 0.0686). Regarding the lengths and diameters of the stents used to vascularize the MB and SB, they were as follows:

- MB stent diameter ranged from 2.7 to 4 mm (3.52 ± 0.38 mm).
- MB stent length ranged from 18 to 48 mm (32 ± 8 mm).
- SB stent diameter ranged from 2.57 to 3.75 mm (3.29 ± 0.34 mm).
- SB stent diameter ranged from 16 to 48 mm (24 ± 6 mm).

Patients treated by provisional stenting mostly had an additional single-vessel disease (55.63%), unlike most patients treated with the two-stent technique had an additional two-vessel disease (66.67%) (*p*-value 0.02). The distribution of bifurcation was mainly LM-LAD in patients treated by provisional stenting (76.76%), while 90.5% of patients treated with the two-stent technique had a plaque distribution of LM-LAD-LCX (*p*-value < 0.001). The most common medina classification in the provisional stenting group was 1, 1, 0 (52.82%) and 1, 1, 1 (90.48%) in the two-stent technique group (*p*-value < 0.001).

Femoral access was the main access used in both groups and the most encountered vascular access complication encountered was hematomas (Table 4).

**Table 4:** Vascular access and vascular access complications

*Vascular access*					
Femoral	99 (69.72%)	18 (85.71%)	***X***^**2**^** = **2.311	0.128	NS
Radial	43 (30.28%)	3 (14.29%)			
*Vascular access complications*					
Hematoma	5 (3.52%)	0 (0%)	Fisher's exact test	1.00	NS
Pseudoneurysm	3 (2.11%)	0 (0%)			
Dissection	3 (2.11%)	0 (0%)			

*Chi-square test of significance (*X*^2^)

As regards LAD and LCX preparation by balloon inflation, the LAD was predilated in 76.06% and 90.48% in provisional and two-stent technique groups, respectively, while the LCX was predilated in 23.94% and 90.48% in the aforementioned groups (*p*-value < 0.001). The LAD was stented first in all cases with an intended provisional stenting technique, while the LCX was stented first in all cases with a two-stent technique (*p*-value < 0.001). Rewiring the LCX was needed in 73.24% of cases treated by provisional stenting but it was needed in all cases treated by the two-stent technique (*p*-value 0.004). Kissing balloons after the first stent were used in 69.72% of cases in the provisional technique and 95.24% in the two-stent technique (*p*-value 0.014).

The majority of cases had a TIMI III flow for the SB in both groups (83.1% in provisional stenting and 80.95% in the two-stent technique), the *p*-value was 0.833. Further improvement in the side vessel was needed in 34 (23.94%) of cases with provisional stenting (*p*-value < 0.001). The most likely cause for LCX stenting in the provisional group was the ostial LCX compromise (13.4%), followed by the presence of a residual lesion (7.8%) or due to occurrence of dissection in LCX (2.8%). Kissing balloons after second stenting was done in 19.72% versus 95.24% of cases in provisional and two-stent techniques respectively (*p*-value < 0.001). Final POT was done in 88.73% of cases with provisional stenting and in all cases with two-stent technique (*p*-value 0.229) 0.23.94% of cases that were intended for provisional stenting ended by a two-stent technique (*p*-value < 0.001). Additional vessel stenting was higher with provisional stenting (*p*-value 0.047) and mainly RCA stenting (*p*-value 0.027). GPIIb/IIIa inhibitors were used in17.61% and 4.76% of cases in the 2 techniques, respectively.

Functional lesion assessment was used in only 6.34% of cases treated by provisional stenting.

### Procedural outcomes

The most common complication for patients treated by provisional stenting was cardiac arrest (2.82%) and CIN (3.52%) and cardiac tamponade and dissection (4.76%each) in cases treated by the two-stent technique. Technical success was achieved in 90.14% of cases treated by provisional stenting versus 90.48% in cases treated by the two-stent technique. Angiographic success was achieved in 87.32% of cases treated by provisional stenting versus 90.48% in cases treated by the two-stent technique (Table 5).

**Table 5:** Procedural outcomes

	Intended stenting technique		Test of significance		
	Provisional	Two-stent technique			
	*N* (%)	*N* (%)	Value	*p*-Value	Sig
*Complications*					
Arrest	4 (2.82)	0 (0)	Fisher's exact test	0.296	NS
Cardiogenic shock	5 (3.52)	0 (0)			
Tamponade	0 (0)	1 (4.76)			
Dissection	4 (2.82)	1 (4.76)			
CIN	5 (3.52)	0 (0)			
*Technical success*					
Yes	14 (9.86)	2 (9.52)	Fisher's exact test	1.00	NS
*Angiographic success*					
Yes	124(87.32)	19 (90.48)	Fisher's exact test	1.00	NS

### Medical treatment and intended DAPT duration

Clopidogrel was used more than ticagrelor in both groups (67.61% and 66.67%, respectively) and the same for atorvastatin which was used more than rosuvastatin in both groups as well (75.35% and 61.9%, respectively). Most patients were planned for 12 months of DAPT unless indicated otherwise for specific situations (Table 6).

**Table 6:** Medical treatment and intended DAPT duration

	Intended stenting technique		Test of significance		
	Provisional	Two-stent technique			
	*N* (%)	*N* (%)	Value	*p*-Value	Sig
*P2Y12 type*					
Clopidogrel	96 (67.61)	14 (66.67)	***X***^***2***^** = **0.007	0.932	NS
Ticagrelor	46 (32.39)	7 (33.33)			
*Statins*					
Atorvastatin	107 (75.35)	13 (61.9)	***X***^***2***^** = **1.793	0.192	NS
Rosuvastatin	35 (24.65)	8 (38.1)			
*DAPT intended duration*					
One month	1 (0.7)	0 (0)	Fisher's exact test	0.308	NS
6 months	7 (4.93)	2 (9.52)			
12 months	128 (90.14)	17 (80.95)			
For life	6 (4.23)	2 (9.52)			

*Chi-square test of significance (*X*^2^)

### In-hospital outcomes

In-hospital outcomes failed to show any statistical significance between both groups even though most events were in the provisional stenting group. In-hospital death was encountered in 6.34% of cases undergoing provisional stenting, among which 5.36% were due to a cardiovascular cause. MI was diagnosed in 4.23% of cases treated by provisional stenting. Total in-hospital MACCE was found to be 5.63% in patients treated by provisional stenting and 0% in the two-stent technique (Table 7).

**Table 7:** In-hospital outcomes

	Intended stenting technique		Test of significance		
	Provisional	Two-stent technique			
	*N* (%)	*N* (%)	Value	*p*-Value	Sig
*All-cause mortality*					
Yes	9 (6.34)	0 (0)	Fisher's exact test	0.606	NS
*CV mortality*					
Yes	8 (5.63)	0 (0)	Fisher's exact test	0.598	NS
*Cerebrovascular events*					
Yes	0 (0)	0 (0)			
*MI*					
Yes	6 (4.23)	0 (0)	Fisher's exact test	1.00	NS
*Stent thrombosis*					
Possible	0(0)	0(0)	Fisher's exact test	1.00	NS
Probable	5 (3.52)	0 (0)			
Definite	1 (0.7)	0 (0)			
*MACCE*					
Yes	8 (5.63)	0 (0)	Fisher's exact test	0.598	NS
*EF*					
> 50%	44 (31.88)	3 (14.29)	***X***^***2***^** = **2.993	0.224	NS
< 40%	58 (42.03)	10 (47.62)			
41–49%	36 (26.09)	8 (38.1)			
*TVF*					
Yes	1 (0.71)	0 (0)	Fisher's exact test	1.00	NS

*Chi-square test of significance (*X*^2^)

### 30-day outcomes

The incidence of all-cause mortality at 30 days was 2.26% versus 4.76% in both the provisional stenting and the two-stent groups, respectively. Out of which 0.75% and 4.76% were due to cardiovascular etiology. A single stroke of hemorrhagic etiology occurred in the provisional stenting group. A single case of MI was diagnosed in the provisional stenting group.

Total MACCE was found to be 2.96% in the provisional stenting group versus 4.76% in the two-stent group. Stent thrombosis was probable in 0.75% and 4.76% of both groups, respectively. At 30 days of follow-up, EF in the provisional group was mostly > 50% (37.59%)and mostly < 40% in the two-stent group (57.14%) and that was the only significant outcome at this time interval of follow-up(*p*-value 0.015).TVF was recognized in 1.5% of cases treated by provisional stenting; however, none was detected in the two-stent group (Table 8).

**Table 8:** 30-day outcomes

	Intended stenting technique		Test of significance		
	Provisional	Two-stent technique			
	*N* (%)	*N* (%)	Value	*p*-Value	Sig
*All-cause mortality*					
Yes	3 (2.26)	1 (4.76)	Fisher's exact test	0.447	NS
*CV mortality*					
Yes	1 (0.75)	1 (4.76)	Fisher's exact test	0.255	NS
*Cerebrovascular events*					
ICH	1 (0.75)	0 (0)	Fisher's exact test	1.00	NS
*MI*					
Yes	1 (0.75)	0 (0)	Fisher's exact test	1.00	NS
*MACCE*					
Yes	4(2.96	1(4.76)	Fisher's exact test	0.52	NS
*Stent thrombosis*					
Definite	0(0)	0(0)	Fisher's exact test	0.255	
Possible	0(0)	0(0)			
Probable	1 (0.75)	1 (4.76)			
*EF*					
> 50%	50 (37.59)	3 (14.29)	***X***^***2***^** = **8.367	0.015	S
< 40%	36 (27.07)	12 (57.14)			
41–49%	47 (35.34)	6 (28.57)			
*TVF*					
Yes	2 (1.5)	0 (0)	Fisher's exact test	1.00	NS

*Chi-square test of significance (*X*^2^)

### One-year outcomes: (Fig. 1)

**Fig. 1:**
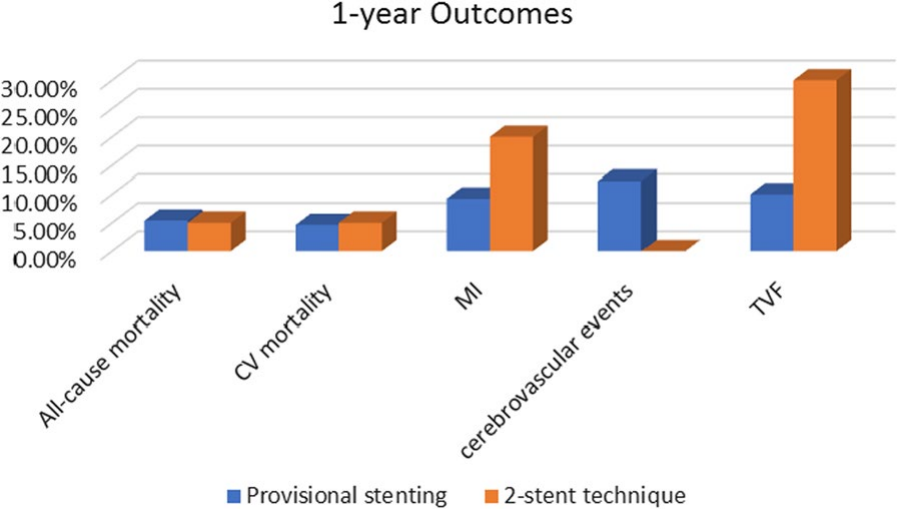
One-year outcomes

Overall MACCE was found to be 22.31% in the provisional group and 30% in the two-stent group (*p*-value0.57). After 1 year of follow-up EF in the provisional group was mostly in the 41–49%range (37.69%)while it was mostly in the < 30% range (60%)in the two-stent group (*p*-value 0.044).

All-cause mortality occurred in 5.38% in the provisional stenting group and 5% in the two-stent technique group. Among these, 4.62% and 5% were due to cardiovascular etiology, respectively. 9.23% of cases treated by provisional stenting suffered TIA and 3.08% suffered an embolic stroke. MI rate in the provisional group was 9.23%, and 30% in the two-stent group (*p*-value 0.17).

TVF was recognized in 10% of cases treated by provisional stenting and 30% of cases treated by two-stent technique (*p*-value 0.023) (Table 9).

**Table 9:** One-year outcomes

	Intended stenting technique		Test of significance		
	Provisional	Two-stent technique			
	*N* (%)	*N* (%)	Value	*p*-Value	Sig
*CV mortality*					
Yes	6 (4.62)	1 (5)	Fisher's exact test	1.00	NS
*All-cause mortality*					
Yes	7 (5.38)	1 (5)	Fisher's exact test	1.00	NS
*Cerebrovascular events*					
TIA	12 (9.23)	0 (0)	Fisher's exact test	0.424	NS
Embolic	4 (3.08)	0 (0)			
*MI*					
Yes	12 (9.23)	6 (30)	Fisher's exact test	0.017	S
*MACCE*					
Yes	29 (22.31)	6 (30)	Fisher's exact test	0.57	NS
*Stent thrombosis*					
Probable	4 (3.08)	0 (0)	Fisher's exact test	1.00	NS
Possible	5 (3.85)	0 (0)			
*EF*					
> 50%	40 (30.77)	3 (15)	***X***^***2***^** = **6.253	0.044	S
< 40%	41 (31.54)	12 (60)			
41–49%	49 (37.69)	5 (25)			
*TVF*					
Yes	13 (10)	6 (30)	Fisher's exact test	0.023	S

*Chi-square test of significance (X^2^)

## Discussion

In a single-center study for LM disease, in the period between May 1 2020 and April 30, 2021, 163 patients were included. All patients underwent PCI using a newer generation of DES. We found that LM PCI as a revascularization approach in symptomatic patients can be adopted with low one-year adverse event rates, whether adopting the provisional approach or the intended two-stent technique. That was a similar conclusion to previous studies as the EBC Main and DK-crush-V trials [19, 22].

At 30 days follow-up, this study showed no statistically significant difference between the provisional and two-stent group regarding MACE, stent thrombosis, or TVF, unlike the DK-crush-V randomized trial [27] which was able to show a significantly higher incidence of cardiac death (1.7% vs 0%, *p*-value 0.046) and probable stent thrombosis (2.1% vs. 0%, *p*-value 0.04) in the provisional stenting group at 30-days follow-up.

This study was able to come up with a statistically significant difference in one-year MI (9.23% vs 30%, *p*-value 0.017) and one-year TVF (10% vs 30%, *p*-value 0.023) which were higher in the two-stenting technique. EBC Main [22] however couldn't show statistically significant difference between both groups in outcomes at one-year follow-up. On the contrary, the DK-crush-V trial showed a higher incidence of probable stent thrombosis (2.5% vs 0%, *p*-value 0.03) in the provisional stenting group at one-year follow-up. Miroslaw Ferenc et al. [13] showed a significantly higher incidence of TVF (17.4% vs 27.2% *p*-value < 0.01) and MACE (41.5% vs 49%, *p*-value 0.03) in the two-stent technique group more than the provisional stenting group but that was at ten-year follow-up.

The discrepancy in results between this study and others is probably due to one of many reasons. At first, The DK-crush-V study used cardiac death and target vessel-related myocardial infarction rather than death and myocardial infarction and this would reduce the overall number of events. Secondly, the coronary anatomy was different. The respective SYNTAX scores were 31 (DK crush) versus 23 (EBC MAIN) and the side-vessel lesion lengths were 16 mm (DK crush) versus 7 mm (EBC MAIN—although the measurement methodology may have differed between the studies). Therefore, the extent of disease was greater in the DK-crush-V study and indeed 45% of patients in the provisional group had implantation of two stents versus 22% in EBC MAIN. Thirdly, the philosophical approach varied between the two trials. The DK-crush technique was pioneered by the Chinese Cardiology teams who undertook the DK-crush-V trial, whereas the stepwise provisional approach has been championed by the European Bifurcation Club since its inception. Hence unconscious biases are likely to have played a part in both trials. Attention to detail regarding the specific technical aspects of each procedure likely differed in the two studies, and results may have varied slightly as a result. For example, in the DK-crush-V study, the POT was not described as a part of the procedure after initial stent placement in the main vessel, and therefore wire passage behind stent struts may have occurred in some cases, whereas in EBC MAIN it was required and was undertaken in 85%. It is of note that the stent thrombosis rate in the two trials was seen to be 2.5% (DK) versus 1.7% (EBC) for the provisional group and 0.4% (DK) versus 1.3% (EBC) for the systematic group. Lastly, this study adopted only the DK-crush technique along with minicrush in a minority of cases when targeting a two-stent strategy from the start, unlike other studies which adopted other techniques. This study showed that the tendency for provisional stenting then bail-out two-stent procedures is the likely habit during PCI to LM bifurcations.

The use of IVUS or FFR in this study was a major limitation (only 6.34%of cases) due to a lack of feasibility on multiple occasions compared to other studies where the use of adjunctive assessment was higher (around 40% in DK-crush-V and 30% in EBC MAIN). However, that didn't much alter the outcomes between this study and others. That was probably due to the tendency to resort to provisional stenting when an adjunctive assessment wasn't available and the comparable rates of technical and angiographic success compared to other studies.

The mean age was 59 years in this study. It was a younger range than the EBC MAIN and DK-crush-V trials where the mean age was 70.2 years and 64 years, respectively. Patients in this study presented at an earlier age probably due to the higher prevalence of risk factors such as DM and obesity among the Egyptian population.

The most common presentation in the provisional stenting group was CCS, and in the two-stent technique, it was NSTEMI (23.24% and 42.86%, *p*-value 0.036). EBC MAIN's candidates were chronic coronary syndrome patients mostly in the two groups. Miroslaw Ferenc et al. had the most common presentation in both arms for patients presenting with chronic coronary syndrome (*p*-value 0.54). Unstable angina was the most common presentation in the DK-crush-V trial (*p*-value 0.49).

A majority of cases had two-vessel disease, mainly LM-LAD in the provisional stenting arm and 3 vessel disease, mainly LM, LAD and LCX in cases treated by the two-stent technique (55.63% and 66.67%, *p*-value 0.02). Miroslaw Ferenc et al. included patients mostly with three-vessel disease.

The most frequently encountered Medina classes among both groups were 1, 1, 1 and 1, 1, 0 and 1, 0, 1 (*p*-value < 0.001), which was concordant with Miroslaw Ferenc et al. (*p*-value < 0.001), EBC Main and DK-crush-V trial which showed similar results in the 1, 1, 1 medina classification percent.

There was a tendency for SB preparation in the double stenting arm in concordance with the DK-crush-V trial (*p*-value < 0.001) and EBC MAIN. In this study, MB was stented first most of the time unless it was a case of DK-crush stenting (*p*-value < 0.001). These steps in the study were concordant with EBC Main.

In the case of provisional stenting, results of TIMI flow in SB were adequate in more than 80% of cases but that was considered a little less outcome than EBC Main and DK-crush-V trial. Rewiring the SB followed by kissing was a common step in the procedures in concordance with EBC Main in cases of provisional stenting.

Further need for second stenting was needed in almost a quarter of cases (*p*-value < 0.001) similar to EBC Main. The most common reason for stenting the SB was the fact that there was a significant lesion requiring improvement (*p*-value < 0.001) like EBC MAIN and Miroslaw Ferenc et al. Incidence of dissection in SB was significantly higher in EBC MAIN than in this study (2.82% vs 10%, respectively).

Final POT was done in 90.2% of cases, a similarly high percentage as in EBC MAIN and DK-crush-V. Final balloon kissing was a usual step in both groups like the DK-crush-V trial. Although 87.1% of cases were intended for provisional stenting technique, 20.9% of the total cases needed bail-out second stenting by TAP. Final kissing after the second stent was done at equal rates as in EBC Main.

The most used two-stent techniques were the TAP technique followed by DK crush (*p*-value < 0.001). EBC MAIN's most commonly used technique was the culotte technique followed by the TAP technique. Miroslaw Ferenc et al. adopted two-stenting techniques, TAP and culotte stenting for the bail-out strategy. It was noticed that this study didn't collect a diversity of cases with stenting techniques such as culotte stenting and that was probably because of the unfavorable anatomy of vessels for revascularization by this technique, especially in absence of adjunctive assessment by IVUS or FFR in addition to operators' preferences.

Syntax score tended to be higher in the two-stent technique arm of the study (*p*-value < 0.001) discordantly with EBC MAIN and DK-crush-V trials where SYNTAX score was equally distributed among the 2 groups.

Additional vessel stenting was statistically significantly higher with provisional stenting (*p*-value < 0.001) and that was discordant with EBC MAIN where it was higher with two-stent and DK-crush-V trial where it was equal between the two groups with no statistical significance in both. This could be explained by the tendency to refer patients with higher SYNTAX scores for CABG whenever the complexity of coronary anatomy rises.

Femoral access was the most used access (69.72% in provisional stenting and 85.71% in the two-stent technique) unlike EBC MAIN and DK-crush-V trials and the most frequently encountered problem was access hematomas. This is due to a combination of lack of equipment for radial access on some occasions and the operator's preference and experience as well.

Probable stent thrombosis was higher in the provisional stenting group similar to EBC MAIN and to DK-crush-V trial where it was of statistical significance. It is of note that the stent thrombosis rate in the two trials was seen to be 2.5% (DK-crush-V) versus 1.7% (EBC MAIN) for the provisional group and 0.4% (DK) versus 1.3% (EBC) for the systematic group. Stent thrombosis rates were low in both groups. The two-stent strategy has frequently been associated with higher stent thrombosis rates in the literature; however, in both the DK-crush-V and EBC MAIN studies, stent thrombosis rates at 1 year were low and undifferentiated between the two groups. The low incidence of stent thrombosis is reassuring and may reflect thinner-strut second and third-generation stents along with an improved understanding of optimal implantation characteristics.

## Study limitations

Limited sample size and only one-year follow-up were the major limitations of this study. Also, the limited use of adjunctive techniques to assess LM artery lesions and to guide procedures such as IVUS, FFR, or OCT was very limited due to their lack of feasibility.

## Conclusions

LMCA lesions treatment by PCI is considered a safe and beneficial solution. Provisional stenting is the preferred approach bearing in mind that bail-out procedures may be sought in case the SB needs further treatment. Adjunctive assessment by IVUS or FFR may help achieve better outcomes and efforts should be performed to facilitate their feasibility.

## Data Availability

The datasets used and analyzed during the current study are available from the corresponding author on reasonable request.

## Abbreviations

AF: Atrial fibrillation
BMI: Body mass index
CABG: Coronary artery bypass grafting
CCS: Chronic coronary syndrome
CHB: Complete heart block
CIN: Contrast-induced nephropathy
CKD: Chronic kidney disease
CLD: Chronic lung disease
cTn.: Cardiac troponin
DES: Drug-eluting stent
DM: Diabetes mellitus
ECG: Electrocardiogram
EF: Ejection fraction
HTN: Hypertension
ICH: Intra-cranial hemorrhage
LAD: Left anterior descending
LCX: Left circumflex
LDL: Low-density lipoprotein
LM: Left main
LMCA: Left main coronary artery
MB: Main branch
MI: Myocardial infarction
NSTE-ACS: Non-ST-elevation acute coronary syndrome
NSTEMI: Non-ST-elevation myocardial infarction
PAD: Peripheral arterial disease
PCI: Percutaneous coronary intervention
POT: Proximal optimization technique
RCA: Right coronary artery
SB: Side branch
STEMI: ST-elevation myocardial infarction
TIA: Transient ischemic attack
TVF: Target vessel failure
UA: Unstable angina
URL: Upper reference limit

## References

[1] Collet C, Capodanno D, Onuma Y (2018). Left main coronary artery disease: Pathophysiology, diagnosis, and treatment. Nat Rev Cardiol.

[2] Smith S (2001). American College of Cardiology/American Heart Association task force on practice guidelines (Committee to revise the 1993 guidelines for percutaneous transluminal coronary angioplasty) ; Society for Cardiac Angiography and Interventions : ACC/AHA guidelines for percutaneous coronary intervention (revision of the 1993 PTCA guidelines)-executive summary : a report of the American College of Cardiology/American Heart Association task force on practice guidelines (Committee to revise the 1993 guidel. Circulation.

[3] Huang HW, Brent BN, Shaw RE (2006). Trends in percutaneous versus surgical revascularization of unprotected left main coronary stenosis in the drug-eluting stent era: a report from the American College of Cardiology-National Cardiovascular Data Registry (ACC-NCDR). Catheter Cardiovasc Interv.

[4] Kolh P, Windecker S, Alfonso F (2014). 2014 ESC/EACTS guidelines on myocardial revascularization. Eur J Cardiothorac Surg.

[5] Chieffo A, Tanaka A, Giustino G, et al (2017) The DELTA 2 Registry A multicenter registry evaluating percutaneous coronary intervention with new-generation drug-eluting stents in patients with obstructive left main coronary artery disease 10.1016/j.jcin.2017.08.050 29217002

[6] Rab T, Sheiban I, Louvard Y, Sawaya FJ, Zhang JJ, Chen SL (2017). STATE-OF-THE-ART REVIEW current interventions for the left main bifurcation. JACC Cardiovasc Interv.

[7] Chieffo A, Magni V, Latib A (2010). 5-year outcomes following percutaneous coronary intervention with drug-eluting stent implantation versus coronary artery bypass graft for unprotected left main coronary artery lesions: the Milan experience. JACC Cardiovasc Interv.

[8] Lee JY, Park DW, Yun SC (2009). Long-term clinical outcomes of sirolimus- versus paclitaxel-eluting stents for patients with unprotected left main coronary artery disease. Analysis of the main-compare (revascularization for unprotected left main coronary artery stenosis: comparison of percutaneous coronary angioplasty versus surgical revascularization) registry. J Am Coll Cardiol.

[9] Migliorini A, Valenti R, Parodi G (2016). Angiographic and clinical outcomes after everolimus-eluting stenting for unprotected left main disease and high anatomic coronary complexity. JACC Cardiovasc Interv.

[10] Meliga E, Garcia-Garcia HM, Valgimigli M (2008). Longest available clinical outcomes after drug-eluting stent implantation for unprotected left main coronary artery disease: the DELFT (Drug Eluting stent for LeFT main) registry. J Am Coll Cardiol.

[11] Takagi K, Ielasi A, Chieffo A (2013). Impact of residual chronic total occlusion of right coronary artery on the long-term outcome in patients treated for unprotected left main disease. Circ Cardiovasc Interv.

[12] Steigen TK, Maeng M, Wiseth R (2006). Randomized study on simple versus complex stenting of coronary artery bifurcation lesions: the nordic bifurcation study. Circulation.

[13] Ferenc M, Banholzer N, Hochholzer W (2019). Long-term results after PCI of unprotected distal left main coronary artery stenosis: the Bifurcations Bad Krozingen (BBK)-Left Main Registry. Clin Res Cardiol.

[14] Hildick-Smith D, de Belder AJ, Cooter N (2010). Randomized trial of simple versus complex drug-eluting stenting for bifurcation lesions. Circulation.

[15] Behan MW, Holm NR, de Belder AJ (2016). Coronary bifurcation lesions treated with simple or complex stenting: 5-year survival from patient-level pooled analysis of the Nordic Bifurcation Study and the British Bifurcation Coronary Study. Eur Heart J.

[16] Cho S, Kang TS, Kim JS (2018). Long-term clinical outcomes and optimal stent strategy in left main coronary bifurcation stenting. JACC Cardiovas Interv.

[17] Kim WJ, Kim YH, Park DW (2011). Comparison of single-versus two-stent techniques in treatment of unprotected left main coronary bifurcation disease. Catheter Cardiovas Interv.

[18] Choi KH, Song YB, Lee JM (2020). Prognostic effects of treatment strategies for left main versus non-left main bifurcation percutaneous coronary intervention with current-generation drug-eluting stent. Circ Cardiovasc Interv.

[19] Chen SL, Zhang JJ, Han Y, et al (2017) Double kissing crush versus provisional stenting for left main distal bifurcation lesions DKCRUSH-V Randomized Trial

[20] Zhang JJ, Ye F, Xu K (2020). Multicentre, randomized comparison of two-stent and provisional stenting techniques in patients with complex coronary bifurcation lesions: the definition II trial. Eur Heart J.

[21] Burzotta F, Dzavik V, Ferenc M, Trani C, Stankovic G (2015). Technical aspects of the T And small Protrusion (TAP) technique. EuroIntervention.

[22] Hildick-Smith D, Egred M, Banning A (2021). The European bifurcation club left main coronary stent study: a randomized comparison of stepwise provisional vs. systematic dual stenting strategies (EBC MAIN). Eur Heart J.

[23] Zhang JJ, Chen SL (2015). Classic crush and DK crush stenting techniques. EuroIntervention.

[24] Galassi AR, Tomasello S, Sacchetta G, Seminara D, Canonico L, Tamburino C (2008). The mini-crush technique for the treatment of coronary trifurcation lesions. EuroIntervention.

[25] Kristian T, Alpert JS, Jaffe AS (2018). Fourth universal definition of myocardial infarction. J Am Coll Cardiol.

[26] Geyer M, Wild J, Hirschmann M (2020). Predictors for target vessel failure after recanalization of chronic total occlusions in patients undergoing surveillance coronary angiography. J Clin Med.

[27] Chen X, Li X, Zhang JJ (2019). 3-Year outcomes of the DKCRUSH-V trial comparing DK crush with provisional stenting for left main bifurcation lesions. JACC Cardiovas Interv.

